# Retraction Note: Caveolin-1 promotes invasion and metastasis by upregulating Pofut1 expression in mouse hepatocellular carcinoma

**DOI:** 10.1038/s41419-024-06529-5

**Published:** 2024-02-12

**Authors:** Cheng Zhang, Huang Huang, Junshi Zhang, Qiong Wu, Xixi Chen, Tianmiao Huang, Wenli Li, Yubo Liu, Jianing Zhang

**Affiliations:** 1 School of Life Science & Medicine, Dalian University of Technology, Panjin, China; 2 School of Life Science & Biotechnology, Dalian University of Technology, Dalian, China

Retraction to: *Cell Death and Disease* 10.1038/s41419-019-1703-1, published online 17 June 2019

The Editors have retracted this article because there are errors in the western blot bands of Hca-F/p-p38 in Fig. 3d and a duplication of the WT NICD and HEY1 expression panels in Fig. 5c. Jianing Zhang agrees with this retraction. Cheng Zhang, Huang Huang, Junshi Zhang, Qiong Wu, Xixi Chen, Tianmiao Huang, Wenli Li and Yubo Liu have not responded to correspondence about this retraction.

